# Laparoscopic versus open appendicectomy: An Indian perspective

**DOI:** 10.4103/0972-9941.15241

**Published:** 2005-03

**Authors:** Utpal De

**Affiliations:** Department of Surgery, Burdwan Medical College, Burdwan. West Bengal - 713101, India

**Keywords:** Appendicectomy, Open, Laparoscopic

## Abstract

**Background:**

Laparoscopic appendicectomy though widely practiced has not gained universal approval. Laparoscopic appendicectomy in India is relatively new and the literature is scant. This study was aimed to compare laparoscopic with open appendicectomy and ascertain the therapeutic benefit, if any, in the overall management of acute appendicitis.

**Materials and Methods:**

The study group consisted of two hundred and seventy nine patients suffering from acute appendicitis. One hundred patients underwent laparoscopic appendicectomy (LA) and one hundred seventy nine patients underwent open appendecectomy (OA). Comparison was based on length of hospital stay, operating time, postoperative morbidity, duration of convalescence and operative cost in terms of their medians. The Mann-Whitney statistics (T) were calculated and because of large samples, the normal deviate test (Z) was used.

**Results:**

Of the hundred patients, six patients (6%) had the procedure converted to open surgery. The rate of infections and overall complications (LA: 15%, OA: 31.8%, *P* < 0.001) were significantly lower in patients undergoing LA. The median length of stay was significantly shorter after LA (3 days after LA, 5 days after OA, *P* < 0.0001) than after OA. The operating time was shorter {OA: 25 min (median), LA: 28 min (median), 0.01< *P* < 0.05} in patients undergoing open appendicectomy compared to laparoscopic appendicectomy.

**Conclusion:**

Hospital stay for LA is significantly shorter and the one-time operative charges appear to be almost the same. LA is also associated with increased clinical comfort in terms of fewer wound infections, faster recovery, earlier return to work and improved cosmesis.

## INTRODUCTION

The treatment of acute appendicitis remained essentially unchanged since its first description by Charles Mc’burney in 1889 before the New York surgical society.[1] Appendicectomy by Mc’burneys incision remained the procedure of choice for nearly a century until 1983 when Kurt Semm offered an alternative, “laparoscopic appendicectomy”.[2] But as McBurney”s operation is well tolerated with less co-morbidity the benefits of laparoscopic appendicectomy have been difficult to establish. The putative advantages of the laparoscopic approach are quicker and less painful recovery, fewer postoperative complications and better cosmesis.[2] It allows better assessment of other intra-abdominal pathologies. But because the validity of these point’s remains unconvincing and also because of shortage of laparoscopic sets in some hospitals, laparoscopic appendicectomy is not practiced widely. Twenty years later laparoscopic appendicectomy is all set to become the choice of therapeutic modality.[1] This prospective study highlights the advantages of this procedure and proves it to be superior over open appendicectomy.

## MATERIAL AND METHODS

This prospective study was carried out over a twelve-month period and included patients with the clinical diagnosis of acute appendicitis. The patients were divided into two groups. The first group included patients undergoing laparoscopic appendicectomy (LA) and the second group included patients undergoing open appendicectomy (OA). The groups were divided based on the choice of the operative procedure the patients preferred. The patients were explained in details about both the operative procedures and were asked to choose which operative procedure the patient liked to undergo. For patients below fifteen years of age, patient’s relatives were explained about the operative procedure and their choice was considered. In the true sense it is not a randomized control trial, because patients voluntarily opting for the method were chosen.

Those patients who chose to undergo laparoscopic appendicectomy but, had contraindications, i.e ASA IV and physiologically compromised, having to creation of carbon dioxide were excluded from the study.

One hundred patients consented to undergo laparoscopic appendicectomy and one hundred seventy nine patients preferred open appendicectomy. A thorough history regarding onset of pain, radiation, anorexia, vomiting and fever was noted. In females of child bearing age (14 to 44 years) a detailed account of menstrual history was noted to exclude pelvic inflammatory disease. All male patients with right iliac fossa pain; a history of burning sensation during micturation and or hamaturia was noted to exclude the diagnosis of ureteric colic. General survey was performed with special emphasis on recording of pulse, temperature and blood pressure. Abdominal examination to note Mc’burneys tenderness, psoas test, obturator test, cough sign, pain on straight leg rising, localized rigidity of right iliac fossa and rebound tenderness was performed. Per rectal examination was mandatory in all the patients. Other systems were examined to note signs of sepsis. After having diagnosed the patient provisionally as a case of appendicitis further examination to confirm the diagnosis included total count to note leucocytosis, biochemical examination to note blood sugar, urea and creatinine, straight X ray abdomen and ultra sonography. A final decision regarding operative intervention was made for all cases of appendicitis. The patients were explained in details about the operative modalities (laparoscopic and open appendicectomies). They were then given the choice of the operative procedure they would like to undergo.

Open appendicectomy was performed through a Mc’ Burney’s muscle splitting incision. The base of the appendix was left uninvaginated.

For laparoscopic appendicectomy, two-hand laparoscopic appendicectomy using three ports, umbilical (10 mm), suprapubic (5 mm) and right iliac fossa (5 mm) was performed. The appendicular artery was dissected and divided between haemostatic clips. The appendix was secured at the base with three loop ligatures, divided between the two distal ligatures, and removed through the 10 mm umbilical port. The base of the appendix was not invaginated.

Laparoscopy was converted to open appendicectomy if technical difficulties, uncertain anatomy or bleeding was encountered. Peroperatively a note was made as to the macroscopical nature of the appendix. The resected appendix was routinely sent for histopathological examination. In patients with perforation and peritonitis, drainage with No 14 F Ryle’s tube was inserted through the right iliac fossa port.

Post operatively intravenous fluids (IVF) were continued for four hours in patients with uncomplicated appendicitis and normal diet instituted thereafter. For complicated cases (patients with perforation and peritonitis) IVF was continued till normal bowel function returned (return of bowel sounds and passage of flatus). Antibiotic prophylaxis included a single dose of third generation cephalosporin for uncomplicated cases. For complicated cases a third generation cephalosporin along with metronidazole preoperatively at induction and another after twelve hours was given.

Analgesics in the form of Diclofenac sodium injections were given for twenty-four hours. Further analgesics were given based on patients’ perception of pain. Drain was removed when drainage was less than 30 ml in twenty-four hours.

As in comparable series the operating time, length of hospital stay, analgesic requirements, return to full activity and operative cost was recorded. Patients in both the study group were discharged as soon as possible, i.e. when fully mobilized without the need for assistance from attendants to secure personal hygiene was no longer required. They were encouraged to resume normal activity and work as soon as they felt fit. Normal activity was defined as return to usual activity of domestic and social life at the discretion of the patient.

The patients were followed-up monthly for three months three monthly for six months and yearly for two years. Any patients having complications were admitted through emergency and investigated with hematological examination and ultrasonography of the abdomen.

Differences between laparoscopic and open procedures with respect to predictor variables were tested using Z-test after determining the Mann-Whitney statistics (T) in each variable. Hypothesis of interest is H_0_: Mx ≥My against H1: Mx < My, where Mx is the median of a population of LA patients and My is the median of a population of OA patients.

The Mann-Whitney statistic is T= S- n_1_ (n_1_+1)/2,

The test criterion is Z= T-n_1_n_2_/2_______,

√n_1_n_2_ (n_1_+n_2_+1)/12

S= Rank sum of LA patients data, n_1_= total sample of LA patient and n_2_= total sample of OA patients.

## RESULTS

Two hundred seventy nine patients were included in the study of which one hundred patients underwent laparoscopic appendicectomy (35.8%) while one hundred seventy- nine patients underwent open appendicectomy (64.1%).

Eight patients were excluded from the study because of contraindication to creation of carbon dioxide pneumo-peritoneum. Of these 4 patients were ASA grade IV, 2 patients suffering from COPD with decreased TLC, VC and FEV_1_ and 2 patients with persistent hypertension (systolic > 200 mm Hg and diastolic > 110 mm Hg) even on antihypertensive treatment.

Patients were on average 24.7 years old and ranged from 6 years to 77 years. Patients who underwent LA were older (LA: 25.1 years, OA: 24.3 years) and more likely male (LA: 61% male, OA: 58.1% male). A large percentage (30.1%) of the lower and middle-income group preferred laparoscopic appendicectomy contrary to the popular belief that laparoscopic surgery was a delicacy for the higher income group (5.7%). (Table 1)

**Table 1:** Demographic profile of the patients

Variables	Open appendecectomy (n = 179)	Lap appendecectomy (n = 100)
Mean Age (years)	24.3 years	25.1 years
	(6-77 yrs)	(8-75 yrs)
Sex ratio (F : M)	75: 104	39: 61
Socio-economic status	1213919	394516
(Average Income in Rs/- per month)		
Lower (< 500) Middle (500-5000)		
Higher (> 5000)		

Laparoscopic findings of the hundred patients undergoing laparoscopic appendicectomy are depicted in Table 2. Of the hundred patients subjected to laparoscopy the procedure was successfully completed in 94 patients, while six patients (6%) had the procedure converted to open surgery. Reasons for conversion were difficulty in visualization and dissection of appendix in 3 patients, peritonitis, abscess and perforation contributed in 3 patients.

**Table 2:** Pathology of appendix as noted during operation

Pathology	LA (n = 100)	OA (n = 179)
Inflamed Appendix	60	90
Adhesion	11	5
Lump	5	15
Kink	8	16
Distended Appendix	7	10
Appendicular Perforation	9	43

In the patients who had undergone laparoscopic appendicectomy, inflamed appendix was detected in 89 patients whereas in 5 patients the appendix appeared normal laparoscopically despite severe symptoms. In all these patients the adenexa was normal.

Appendicular lump found in 5 patients in the laparoscopic group were early lumps, not apparent on clinical examination either preoperatively or under anesthesia. In all these patients the appendix was gently dissected with the tip of the sucker nozzle and by hydro-dissection. Caution was also taken during ligation of the base which was friable and tended to cut through.

Fifteen patients in the open group had appendicular lump. Twelve of these were apparent on clinical examination and the criteria for operation were: (a) lump less than 4 cms, (b) mobile lump confined to the right iliac fossa, and (c) time period less than 48 hrs. For the rest 3 patients lump was detected per-operatively and the same principles as for laparoscopic approach were followed.

Length of hospital stay ranged from 2 days to 9 days. The length of stay was significantly shorter after LA (3 days after LA, 5 days after OA, *P* < 0.0001).

The rate of infections and overall complications (LA: 15%, OA: 31.8%, *P* < 0.0001) were significantly lower in patients undergoing LA (Table 3).

**Table 3:** Post-operative complications

Complications	OA (%) (n=179)	LA (%) (n=100)
Wound/Port Infection	25 (14)	4 (4)
Fistula	4 (2.2)	2 (2)
Late Intestinal Obstruction	2 (1.1)	1 (1)
Incisional / Port site Hernia	17 (9.5)	2 (2)
Secondary Hemorrhage / Bleeding from port	2 (1.1)	5 (5)
Injury To Other Organs	7 (3.9)	1 (1)

There was an insignificantly shorter operating time {OA: 25 min (median), LA: 30 mins (median), 0.05> *P* > 0.01} in patients undergoing open appendicectomy compared to laparoscopic appendicectomy.

Histopathology revealed normal appendix in 5 patients (5%) in the LA group and 27 patients (15.08%) in the OA group. For the other patients in both the groups histopathology was suggestive of acute appendicitis.

After comparing other covariates (Table 4), LA remained associated with fewer days return to general diet, shorter duration of parenteral analgesia, fewer milligrams of oral analgesia, a shorter postoperative hospital stays and earlier return to full activity.

However the billed charges and direct costs appeared to be marginally higher for laparoscopic appendicectomy but the total cost was less given the shorter hospital stay and abbreviated recuperative period.

**Table 4:** Comparison of major parameters of the study

Variables	LA Median	OA Median	Mann-Whitney (T)	Normal Deviate *(Z)*	*P value*
Admission to operation (days)	4	2	15115	10	<0.0001
Parenteral analgesia (days)	1	5	318	13	<0.0001
Oralanalgesia (days)	1	7	8	14	<0.0001
NPO to general diet (days)	1	3	26	14	<0.0001
Operative time (mins)	30	25	9946	2	0.01<P>0.05
Hospital stay (days)	3	5	4098	8	<0.0001
To full activity (days)	3	14	0	14	<0.0001
Operative cost (INR)	925	923	9387	0.7	>0.3

**LA (n = 100); OA (n = 179).

## DISCUSSION

The results of the present study are in keeping with several previous studies where laparoscopic appendicectomy has been shown to be both feasible and safe in comparison with open appendicectomy.[1–12] In addition to improved diagnostic accuracy, laparoscopic appendecectomy confers advantages in terms of fewer wound infections,[3] less pain, faster recovery and earlier return to work.[4] However laparoscopic appendicectomy is time consuming.[5,6] It is also argued that the advantages of laparoscopic appendicectomy are marginal compared to open appendicectomy performed by an experienced surgeon through a short, cosmetically acceptable incision with minimal complication and shorter hospital stay.[2–7]

The question of whether laparoscopic appendicectomy decreases the length of hospitalization has been a matter of great debate over the past decade.[5–9] The literature provides contradictory results. Most studies report a median hospital stay of 2-5 days irrespective of laparoscopic or open procedure. Although some recent retrospective cohort studies or chart reviews found laparoscopic appendicectomy associated with significantly shorter hospital stay,[10–12] Other retrospective investigations reported nonsignificant differences.[13–15] Similarly, some randomized controlled trials associated laparoscopic appendicectomy with decreased hospital stay.[7,9,10–15] However, others report no significant difference between laparoscopic appendicectomy and open appendicectomy.[14,15] Even meta-analyses report controversial findings. Sauerland and associates summarized the results of 28 randomized controlled trials and almost 3000 patients and reported a significant decrease in length of hospital stay in patients undergoing LA.[14] Similar results were found by Golub and colleagues,[14] whereas another meta-analysis failed to show a statistically significant difference in length of hospital stay between LA and OA.[12–14] The heterogeneity of published results regarding length of hospital stay may be caused by a variety of factors: The current literature describes that the difference may be affected by hospital factors[14,15] or social habits,[15] rather than reflecting differences resulting from the operative technique itself. Moreover, further discrepancies may arise from diverse health care policies in different countries. The present study revealed a significantly shorter hospital stay for patients undergoing laparoscopic appendicectomy.

Significant variation in operating time was noted in various controlled studies.[8,10,14] Some studies noted a shorter operating time for patients undergoing open appendicectomy while others revealed no difference. In the present study more operating time (0.05> *P* > 0.01) was noted for laparoscopic appendicectomy. This was because of the learning curve during the earlier phase of our study. Level of surgical experience, patient selection and increased conversion rate in the earlier stages accounted for increased operative time. Later phase of our study revealed a more or less similar operating time for open and laparoscopic appendicectomy.

In accordance with other studies there were significantly fewer wound infections in the laparoscopy group.[4–10] A reduction in wound infection can be achieved by extraction of the specimen through a port or with the use of an endobag, or leaving a non-inflamed appendix in place. This has been confirmed in the present study.

At a glance the median operative cost of laparoscopic appendicectomy seems to be marginally greater (LA:→OA Rs. 925/-: Rs. 923/-) compared to open appendicectomy. But considering the total cost of the disease when cost of accommodation, operation and time of work, daily cost of inpatient unit, hourly cost of operating room- recovery ward and the patients consumption is taken into account laparoscopic appendicectomy provides a clinical comfort and economic benefit in all patients. With the government providing most of the equipment and infrastuctural facility thus curtailing one time operative cost, the economic significance and implications definitely favor laparoscopic appendicectomy. Literatures detailing cost analysis are conflicting and vary according to the standpoint of the disease, the patient, the surgeon, the treatment center, industry and society.[8–15] The cost surplus of the laparoscopic procedure and recovery after surgery were evaluated in these studies, to determine the costs and effects of laparoscopic appendicectomy compared with those of open appendicectomy. A shorter hospital stay, resulting in a marginal difference in itemized total costs between the two procedures, offset the increased operative expenses. The studies concluded that laparoscopic appendicectomy was slightly more expensive, but it allowed earlier return to normal daily activities than open appendicectomy.[1–15]

Because of the competition of laparoscopic and open appendecectomy, open appendecectomy has improved greatly. More and more questions are being raised as to the benefit of laparoscopic appendecectomy. A few recent randomized controlled trials have even gone to the extent questioning the benefits and efficacy of laparoscopic appendicectomy.[14,15] Some authors have concluded once and for all that laparoscopic appendicectomy is out.[15]

But going by our study we definitely find an over all advantage of laparoscopic appendicectomy. Since studies on laparoscopic appendectomies from our country are few, questions remain: Can it be improved any more? Is there a place and need for further randomized controlled trials?
